# Gene Interaction Network Suggests Dioxin Induces a Significant Linkage between Aryl Hydrocarbon Receptor and Retinoic Acid Receptor Beta

**DOI:** 10.1289/txg.7020

**Published:** 2004-06-23

**Authors:** Hiroyoshi Toyoshiba, Takeharu Yamanaka, Hideko Sone, Frederick M. Parham, Nigel J. Walker, Jeanelle Martinez, Christopher J. Portier

**Affiliations:** Laboratory of Computational Biology and Risk Analysis, National Institute of Environmental Health Sciences, Research Triangle Park, North Carolina, USA

**Keywords:** Bayesian networks, dioxin, gene regulatory networks, Markov chain Monte Carlo, retinoic acid receptor, risk assessment, systems biology, toxicogenomics

## Abstract

Gene expression arrays (gene chips) have enabled researchers to roughly quantify the level of mRNA expression for a large number of genes in a single sample. Several methods have been developed for the analysis of gene array data including clustering, outlier detection, and correlation studies. Most of these analyses are aimed at a qualitative identification of what is different between two samples and/or the relationship between two genes. We propose a quantitative, statistically sound methodology for the analysis of gene regulatory networks using gene expression data sets. The method is based on Bayesian networks for direct quantification of gene expression networks. Using the gene expression changes in HPL1A lung airway epithelial cells after exposure to 2,3,7,8-tetrachlorodibenzo-*p*-dioxin at levels of 0.1, 1.0, and 10.0 nM for 24 hr, a gene expression network was hypothesized and analyzed. The method clearly demonstrates support for the assumed network and the hypothesis linking the usual dioxin expression changes to the retinoic acid receptor system. Simulation studies demonstrated the method works well, even for small samples.

Gene expression arrays (gene chips) have enabled researchers to simultaneously monitor the approximate level of mRNA expression for a large number of genes. These mRNA expression levels are one component of the machinery that controls the function and survival of cells; the other components constitute the other major biochemical constituents of a cell such as the actual DNA sequence, protein levels, and cellular substructures. Signal transduction pathways have long been used to describe the sequence of biochemical events that control cellular function and generally include all aspects of the biochemistry of a cell. In the absence of full proteomic data (both primary proteins and modified proteins), it is valuable to understand the quantitative relationship between genes, which we will refer to as gene expression networks. The rates derived from the quantification of gene expression networks provide crude estimates for the overall rates linking genes through complicated signaling pathways. In addition, hypothesized linkages between genes will aid in focusing research efforts in other areas such as proteomics, metabolomics, and toxicologic assays.

We recently used toxicogenomic analysis to examine the response of human peripheral lung epithelial cells to 2,3,7,8-tetrachlorodibenzo-*p*-dioxin (TCDD, dioxin) *in vitro* (Martinez et al. 2002). Exposure to this persistent environmental pollutant has been associated in human populations with increased risk of lung cancer and chronic obstructive pulmonary disease; therefore, understanding its mechanism of action may provide insights into the risk of persistent human exposure not only to TCDD but to other ligands of the aryl hydrocarbon receptor (AhR). In this study we showed a variety of cell-signaling pathways that exhibited a dose-dependent alteration by TCDD. One observation in this study was an alteration in retinoic acid (RA)-responsive genes. Alterations in RA homeostasis have been observed previously in rodents, leading to a retinoid-deficient state. In addition TCDD exposure in rats has been associated with increased incidence of squamous neoplastic and nonneoplastic lesions including squamous cell carcinoma of the lung and hard palate region the oral mucosa (Kociba et al. 1978). Given that alterations in retinoid signaling can affect the differentiation of squamous epithelia, it is possible that the increase in these squamous lesions may be due to a retinoid-deficient state induced by the alteration in retinoid homeostasis.

Identification of the retinoid-responsive genes in the TCDD microarray analyses suggested a functional relationship between AhR activation and retinoid homeostasis and/or signaling in the human lung epithelial cells. Although such relationships can be tested empirically, invariably a large number of functional relationships are possible within a given microarray data set; therefore, priority setting for functional validation studies is often a challenge. In this article we develop a computational approach for evaluating the likelihood that observed changes in gene expression are due to hypothesized functional relationships. We then test the AhR–retinoid interaction using this method.

Several methods have already been proposed for the analysis of gene expression data. The most commonly used methods rely on description of simple fold increases in expression, phylogenetic tree analyses, clustering methods, classification methods, or combinations of these. Methods have also been proposed to develop gene expression networks using dynamical systems defined by ordinary differential equations (Chen et al. 1999), modified linear regression methods (Gardner et al. 2003), Boolean networks (Akutsu et al. 2000) where gene expression data are converted to two states (ON and OFF), discrete networks (Hartemink et al. 2002), and many others. Bayesian networks (Friedman et al. 2000; Pe’er et al. 2001) have been proposed as a means of identifying gene interaction networks (Imoto et al. 2002; Tamayo et al. 1999) and for predicting protein–protein interactions using a combination of different types of genomic data (Jansen 2003). Many of the available methods are discussed in a recent review article (Lockhart and Winzeler 2000). Few methods exist that combine careful statistical estimation and hypothesis testing with quantitative gene interaction models to provide a systems biology–based approach for the analysis of microarray data.

In this article, a Bayesian network approach (Friedman et al. 2000; Imoto et al. 2002) previously suggested is modified to provide direct quantification of gene expression networks using microarray data for a known network. This analytical approach provides a model that can be used for mechanism-based mathematical models and for formal analyses of biological hypotheses.

## Materials and Methods

### Definition of Gene Expression Network

**Figure 1:**
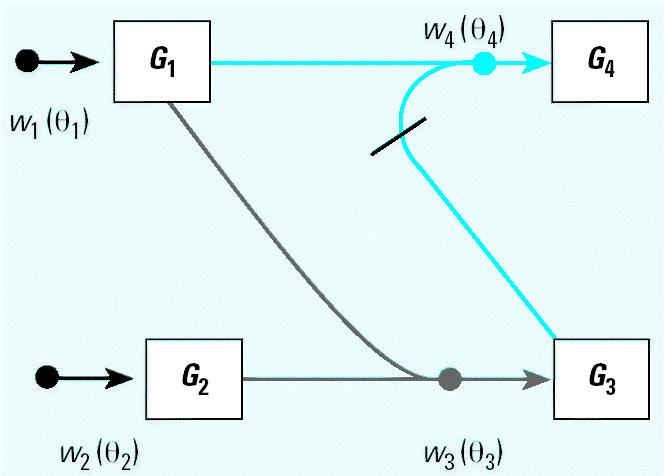
A simple gene expression network consisting of four genes and four nonzero functional relationships.

The basic concept for Bayesian networks in the analysis of gene expression data has been described previously (Friedman et al. 2000; Imoto et al. 2002; Tamada et al. 2003). A gene expression network consists of a collection of *P* genes, denoted by *X*_1_, *X*_2_,…*X_P_*, linked by weighting functions, *w_i_*(θ*_i_*) ( *i* = 1,2,…*P* ), where the subscript **i** denotes that this weighting function pertains to the control of gene *X_i_* by all genes linked to it and θ*_i_* denotes the vector of parameters defining the functional relationship. In cases where the relationship between individual genes is monotonic (i.e., *X_i_* either stimulates or inhibits *X_j_* but cannot have mixed effect), such a network can be easily represented graphically as in Figure 1. Figure 1 is a simple gene expression network consisting of four genes (squares) and four weighting functions (circles), with lines linking the genes and the weighting functions. Two kinds of lines appear in the model. A line with a bar implies inhibition (e.g., gene *X*_3_ inhibits gene *X*_4_ in Figure 1), and a line with no bar implies stimulation (e.g., gene *X*_1_ stimulates gene *X*_4_). No line between genes implies these genes have no direct relationship to each other (*X*_2_ and *X*_4_ are not directly linked). The weighting function combining the effects of genes *X*_1_ and *X*_3_ on gene *X*_4_ is denoted by *w*_4_(θ_4_) in Figure 1.

The vector *W*(θ) = [*w*_1_(θ_1_) *w*_2_(θ_2_)*… w_P_*(θ*_P_*)] fully characterizes the functional relationships between genes in a gene expression network and is the target of any estimation effort to identify and quantify a network. The functional form that can be used for any individual *w_i_*(θ*_i_*) is not restricted. One example is the log-linear gene expression network.

### Log-Linear Gene Expression Network

One of the simplest types of weighting function used to describe a gene expression network is the log-linear weighting function given by the following form:


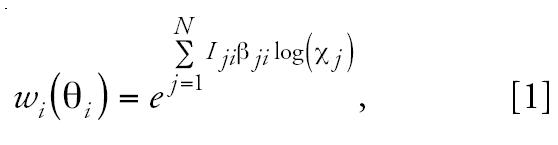


where *x_j_* is the observed level of expression (or ratios of expression) of gene *X_j_*, β*_ji_* is the magnitude by which a change in one log unit of gene *X_j_* will affect the level of expression of gene *X_i_*, and *I_ji_* is an indicator variable describing the direction of the change denoted by β*_ji_*, where *I_ji_* = 1 for stimulation, *I_ji_* = −1 for inhibition, and *I_ji_* = 0 for no effect. For simplicity of notation, we define **B** = [β*_ji_*]*_j_*_= 1,2…_*_p_*_,_ *_i_*_=1,2…_*_p_*, **T** = [*I_ji_*]*_j_*_=1,2…_*_p_*_,_ *_i_*_=1,2…_*_p_*, and **A** = [log(*x*_1_), log(*x*_2_),…log(*x_p_*)], where we refer to **T** as the transition matrix and **B** as the parameter matrix. It is then possible to rewrite Equation 1 in its matrix form given by


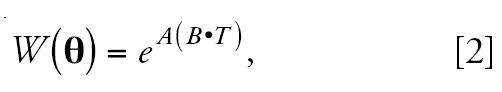


where θ = [β_11_ β_12_*…*β*_pp_*], and the dot represents element-by-element multiplication of **B** and **T**. In the example given by Figure 1, the matrices **B** and **T** are 4 × 4 matrices and have only 4 nonzero elements each [(1,3), (1,4), (2,3), and (3,4)], so the vector of parameters is θ = [β_13_ β_14_ β_23_ β_34_]. Tamada et al. (2003) used a non-parametric B-spline for *w_i_* (θ*_i_*)*.* Such a method could be used in this context as well, where the breakpoints in the splines are at individual doses or times used for an experimental design.

The transition matrix provides the qualitative structure of the gene expression network, and the parameter matrix quantifies the strength of the relationship between the genes. In the following we use *N_p_*(θ) to represent a general gene expression network with *P* genes and *N_TP_*(θ) to specifically represent a log-linear gene expression network with *P* genes.

### Bayesian Network Estimation Procedure

Like any other biological measurement, it can be presumed that two observations taken from seemingly identical examples may differ because of uncontrolled variables or simple random fluctuation; this difference is traditionally defined as random variation about the mean behavior in a model. With random variation, **x** = [*x*_1_*, x*_2_*…x_p_*] is an observation from a random matrix **X** = [*X*_1_*, X*_2_*…X_P_*]. The simplest method by which random variation can be included in a gene interaction network is to assume that *X_i_* is conditional on knowledge of the other *X*’s and θ follows a prescribed probability density function. Define **Xi** **=** [*X*_1_*, X*_2_*,…X_i_*_−1_*, X_i+_*_1_*,…X_P_*] and define *f_i_* (*X_i_|***X̄**_**i**_**,** θ) to be the conditional density of *X_i_*. If a gene has a regulatory effect on gene *X_i_*, that gene is referred to as a “parent of gene *X_i_*”; in other words, it belongs to the set referred to by **Pa**(*X_i_*). Hence, for example, in the model depicted by Figure 1, **Pa**(*X*_3_) = [*X*_1_, *X*_2_]. This notation has been used in other cases and in the context of this modeling, the distribution could then be written as *f_i_* (*X_i_* |**Pa**(*X_i_*)*,* θ). A greater level of statistical complexity is possible by also presuming that the parameters have probability density functions; *h_i_*(θ*_i_*) is referred to as the prior distribution of θ*_i_*. This formulation places the network defined by *N_P_*(θ) and the data into the context of classical Bayesian networks (Jensen 1996).

Suppose that we have *m* sets of microarray data [*x*_1j_*, x*_2_*_j_,…x_Pj_*]*_j_*_= 1,2,_*_…m_* from gene expression network *N_P_*(θ), where individual arrays are independent random samples from the joint density function for the genes. The joint density function for the parameters given the gene expression data, denoted *g* (θ*|* **X** ), is referred to as the posterior distribution and can be estimated using the Markov chain Monte Carlo (MCMC) method (Hastings 1970). In the examples given in this article, the Metropolis algorithm (Andrec and Prestegard 1998) is used to sample from the MCMC to generate samples from the joint density.

### Specific Cases Used in This Analysis

In all analyses that follow, the gene expression network is presumed to be a log-linear network defined by *N_TP_*(θ) in Equations 1 and 2. It is assumed that data arise from microarrays using a relative comparison between two samples (no change results in a value of 1, increased expression > 1, decreased expression < 1), and the distributions for the log of the individual relative gene expression levels conditional on knowledge of **T,** θ and the other *X’*s, *f_i_* (*X̄_i_*|**Xi,** θ), are assumed to be normal, with mean defined as the exponent of *e* in Equation 1 and with standard deviation (SD)σ. All parameters in θ = (**B,S**), where **S** = [σ_1_*,* σ_2_*,…* σ*_P_*] are assumed to have prior distributions (normal for the elements of **B** and uniform for the elements of **S**).

Assume that the structure of **T** (transition matrix) is known without error. In this situation, the qualitative relationship between genes in the gene expression network is known. Taking Figure 1 as an example, expectation of each *log* (*X***i**) (*i* = 1,2,3) becomes *E* [log(*X*_1_)|**T,B,**— **X̄**_**1**_] = 0, *E*[log(*X*_2_)|**T,B,** **X̄**_**2**_] = 0, *E*[log(*X*_3_)|**T,B,** **X̄**_**3**_] = β_13_ log(*X*_1_) + β_23_ log(*X*_2_), and *E*[log(*X*_4_)|**T,B,** **X̄**_**4**_] = β_14_ log(*X*_1_) − β_34_ log(*X*_3_). The ultimate goal of defining a Bayesian network is to derive the posterior distribution for the parameters of interest. To derive the posterior, we must first calculate the conditional likelihood of the data, denoted *L_N_*[**X** |*N_TP_*(θ)]. The likelihood is the product of the individual conditional densities and is written


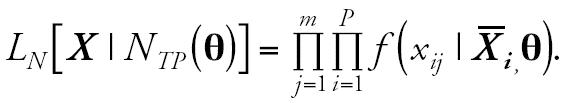


In the MCMC analysis, we must assume a mean and variance for the prior normal distributions for the β’s and bounds on the prior uniform distributions for the σ’s. Several options were chosen for the prior means of the β’s and an uninformative SD (10) was chosen for the prior variance. To develop bounds on the prior uniform distributions for the σ’s, SDs were calculated for each gene across replicates, and the maximum SD observed was multiplied by 2 to set the upper bound, with 0 set as the lower bound. Given these priors and the data, MCMC iterations for each data set analyzed are run until the estimates for the posterior distributions for the β’s and the σ’s are stabilized.

Other distributions and methods could be used to define the priors and generate the posterior distributions for the likelihood and the parameters in the model. In considering a more complicated functional relationship between genes, Michaelis-Menten–type equations could be used to develop networks with restricted maximum and minimum linkages. Such networks would require substantially more data.

A user-friendly software package for these analyses is available from the corresponding author.

### Application to Microarray Gene Expression Data

Martinez et al. (2002) evaluated the change in expression of 2,091 genes in triplicate samples of HPL1A and A549 cells exposed to differing levels (0, 0.1, 1.0, and 10 nM) TCDD for 24 hr. Total RNA was extracted and, using methods described by Martinez et al., hybridized to NIEHS Human ToxChip, version 1.0 (http://dir.niehs.nih.gov/microarray/chips.htm) to obtain changes in gene expression in dioxin-treated cells (one channel) relative to the controls (second channel). They identified 68 genes that were altered in at least one cell line and 15 genes that were altered in both cell lines. Of these, they identified 11 genes that appear to be involved in the effects of TCDD on the retinoid-signaling pathway. In this article we hypothesize a gene interaction network defining the quantitative role of TCDD in altering retinoid signaling based on the current available literature. The data for these 11 genes from the HPL1A cells and the hypothesized network are analyzed using the methods described above.

## Results

### Dioxin Analysis

**Figure 2:**
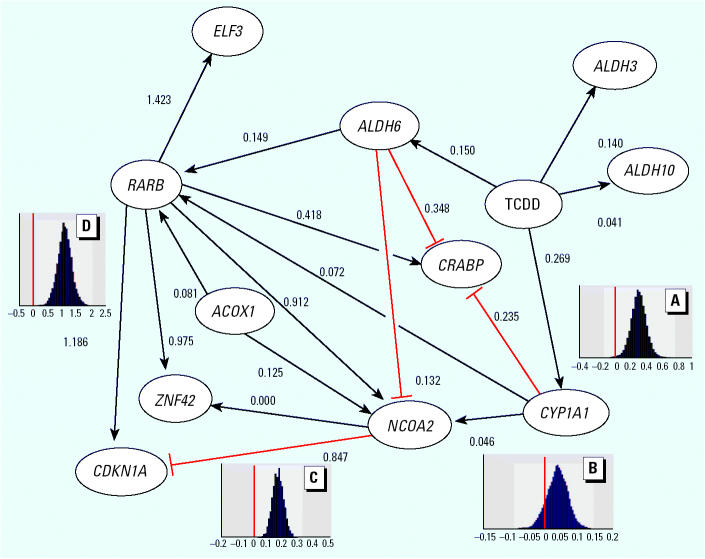
Hypothesized network describing the linkage between AhR-responsive genes and *RARB-*responsive genes, where the numbers represent the mean estimate for the linkage (β) between the two genes on any given line, and the four distributions (*A–D*) are the posterior distributions for the linkages between (*A*) TCDD and *CYP1A1*, (*B*) *CYP1A1* and *NCOA2*, (*C*) *NCOA2* and *CDKN1A*, and (*D*) *RARB* and *CDKN1A*.

2,3,7,8-Tetrachlorodibenzo-*p*-dioxin is a known human carcinogen, a suspected teratogen, and highly toxic in most mammalian species. There has been considerable speculation that TCDD alters the retinoic acid receptor (RAR)–dependent signaling pathway via alteration of the synthesis and metabolism of RA. Microarray data (Martinez et al. 2002) on changes in gene expression in HPL1A lung airway epithelial cells after exposure to TCDD at levels of 0.1, 1.0, and 10.0 nM for 24 hr identified 11 genes with significant changes at the 99% confidence level. The gene identifiers and data are given in Tables 1 and 2. Figure 2 hypothesizes a gene interaction network linking the traditional TCDD-induced genes and genes in the RAR-dependent signaling pathway.

Vitamin A (retinol) is taken up from blood and binds to the *CRBP* in the cytoplasm. Retinol and alcohol dehydrogenases convert the sequestered retinol to retinal, which is then converted to RA by retinal dehydrogenases such as ALDH6 (Rexer et al. 2001). It is also possible that cytochrome P450s such as CYP1A1 may also convert retinal to RA (Zhang et al. 2000). Once RA is synthesized, it binds to cytosolic RA binding proteins (such as CRABP). RA enters the nucleus, where it binds to two types of ligand-activated nuclear transcription factors, the RA receptors (e.g., RARB) and the retinoid X receptors. Several groups have hypothesized that changes observed in RA levels from dioxin exposures are mediated through increased metabolism of retinal to RA through retinal dehydrogenases or cytochrome P450s or both (Schmidt et al. 2003). Using these data together with the known AhR gene battery, we developed a hypothetical gene interaction network (Figure 2).

The predominant linkage to RAR is through upregulation of *ALDH6* and *CYP1A1*, which synthesize RA. TCDD alters the metabolism of all-*trans-*RA (Schmidt et al. 2003), suggesting the linkage between *ALDH6* and *RARB* in Figure 2. *RARB* has been shown to play a role in the inhibition of cellular replication (Sun et al. 2000). *RARB* is assumed to modify the expression levels of four genes: *ELF3*, *NCOA2*, *ZNF42*, and *CDKN1A*. These genes have been shown to be parts of the differentiation pathways of various cell types and are hypothesized to be modified by changes in the RA-signaling pathway. *ELF3* is an epithelial-specific transcriptional regulator that may play a role in lung carcinogenesis (Tymms et al. 1997). *NCOA2*, also known as *GRIP1*, interacts with the five steroid hormone receptor types (Hong et al. 1997; Schmidt et al. 1998). *ZNF42*, also known as *MZF-1*, is a putative transcriptional regulator induced by RA in human myeloid cells (Hromas et al. 1991). *CDKN1A* is induced by RA through *RARB* in human neuroblastoma tumors (Cheung et al. 1998; Liu et al. 1996). Both *ALDH6* and *RARB* affect the regulation of *NCOA2*, which in turn alters the regulation of *ZNF42* and *CDKN1A*. *ACOX1*, the human peroxisomal acylcoenzyme A oxidase, is hypothesized in the model to upregulate both *RARB* and *NCOA2*. *ACOX1* is the first enzyme of the fatty acid beta-oxidation pathway (Varanasi et al. 1994), and changes in this gene are likely to affect endogenous levels of fatty acids known to activate the retinoic X receptor, thereby modulating gene expression (Issemann et al. 1993). The second major linkage occurs between cytochrome P4501A1, *CYP1A1*, and *RARB*. An inducer of *CYP1A1* (β-naphthoflavone) induced the metabolism of all-*trans*-RA in human intestinal epithelial cells (Lampen et al. 2000). *CYP1A1* is upregulated by TCDD (Portier et al. 1993), suggesting the linkage between TCDD and genes in the RAR-signaling pathway such as *RARB*, *NCOA2*, and *CRABP*, a specific carrier protein for vitamin A that influences metabolism of RA and increases the sensitivity of a cell to vitamin A signaling (Boylan and Gudas 1992; Ong 1987). The *CRABP* promoter contains an enhancer region through which RA inhibits *CRABP* transcription (Means et al. 2000).

The slope parameters for all the linkages between genes (β*_ij_* in Equation 1) in Figure 2 were estimated using the Bayesian gene interaction network approach as described above. Prior probability distributions for the log gene expression values were assumed to be normally distributed with a mean of zero and a variance of 1. The SDs (σ_1_, σ_2_, …σ*_P_*) were assumed to have uniform priors ranging from zero to two times the largest SD observed for any one gene (an uninformative prior). A total of 100,000 MCMC samples were obtained, and the last 80% (80,000) were used to estimate the posterior density of the parameters. Although no formal MCMC stopping rule was used, analyses of the last 80,000 MCMC samples clearly supported convergence.

Figure 2 also illustrates the type of results routinely obtained in Bayesian analyses. The four histograms shown in Figure 2 are the empirical posterior densities for 4 of the 19 linkages. The linkage between TCDD and *CYP1A1* (*A*) has a distribution for which there are virtually no values below zero, indicating a strong statistical relationship in these data. The mean value, 0.269, indicates the degree of change in *CYP1A1* expression as a function of the change in TCDD concentration. The means, SDs, and percentages of values below zero are summarized for all 19 linkages in Table 3. The distributions for the variances are presented in Table 4. Other uninformative priors were tried with no significant alteration in the results presented in Table 3. In our Bayesian analysis we estimate the posterior distributions for each parameter given the data. If a distribution for a given parameter has a small probability of being < 0 (such as ≤ 0.1), that parameter supports a linkage between genes.

The network depicted in Figure 2 was developed to test the hypothesis of a linkage between dioxin-responsive genes *CYP1A1* and *ALDH6*, and the RAR-signaling gene *RARB*. The distribution for β*_CYP1A1_*_→_ *_RARB_* had a substantial mass less than zero (26% < 0, Table 3), suggesting a lack of support for the linkage between changes in message for these two genes. Similar results were seen for β*_ALDH6_*_→_ *_RARB_* (20% < 0, Table 3). Examination of the joint density for β*_CYP1A1_*_→_ *_RARB_* and β*_ALDH6_*_→_ *_RARB_* suggested a negative correlation, indicating that the data may not support both linkages simultaneously. This is not surprising, as they are both acting upon the same component of RA synthesis. By forcing β*_CYP1A1_*_→_ *_RARB_* = 0 and again estimating the remaining parameters, we can examine the distribution of β*_ALDH6_*_→_ *_RARB_* under the condition that the other linkage is not present; in this case, β*_ALDH6_*_→_ *_RARB_* had no estimates less than zero (0%) and there was no change in the posterior distribution for the log-likelihood, suggesting almost no change in the fit of the network to the data, even though we dropped the linkage between *CYP1A1* and *RARB*. Conversely, we can set β*_ALDH6_*_→_ *_RARB_* = 0 and examine the distribution of β*_CYP1A1_*_→_ *_RARB_*; here also we see 0% < 0 and no change in the log-likelihood. These two analyses support the hypothesized linkage between TCDD-responsive genes and *RARB*-responsive genes, but only through either *ALDH6* or *CYP1A1*, not both. Finally, setting both β*_CYP1A1_*_→_ *_RARB_* = 0 and β*_ALDH6_*_→_ *_RARB_* = 0 significantly shifts the distribution of the posterior log-likelihood to smaller values (10% reduction overall), suggesting that at least one of these linkages is needed to explain these data.

The only other linkage that did not appear to be supported by these data was the hypothesized linkage between *NCOA2* and *ZNF42*. The distribution for β*_NCOA2_*_→_ *_ZNF42_* had a mean estimate of zero, with 48.5% of the estimates less than zero. Assuming β*_NCOA2_*_→_ *_ZNF42_* = 0 had no impact on the log-likelihood, suggesting this linkage was not needed in the model and that there was no correlation offset with other parameters. Given the sample size and the number of genes in the network, it is surprising that all other linkages appeared to be supported by these data, with the percentage of β values less than zero ranging from 0% for several pairs (β_TCDD →_ *_CYP1A1_*, β*_RARB_*_→_ *_CDKN1A_*, β*_RARB_*_→_ *_ELF3_*, β*_RARB-_*_→_ *_NCOA2_*, β*_RARB_*_→_ *_ZNF42_*, and β*_ACOX1_*_→_ *_NCOA2_*) to 9.4% (β*_ACOX1_*_→_ *_RARB_*).

### Simulation Studies

Although the TCDD example is illustrative of the method, it does not address how well this method works under diverse conditions; this is best addressed by Monte Carlo simulations. One thousand (1,000) simulated experiments from the simple four-gene network in Figure 1 were generated by the computer using sample sizes of 50, 25, and 10 gene chips in each experiment. Twenty-two combinations of the model parameters (θ = [β_13_, β_14_, β_23_, β_34_, σ_1_σ_2_*,* σ_3_*,* σ_4_]) were considered. For each simulation, posterior distributions were calculated and summarized by their means, medians, and SDs. The MCMC process used was identical to that used for the dioxin example, with the exception that only 8,000 iterations of the Metropolis algorithm were performed, and the last 20% (1,600) values were used to calculate the summary statistics. Multiple runs with different starting points were used, with no difference in the final results (not shown).

Table 5 provides representative results from two of the simulation studies. The results indicate that, when sample sizes are sufficiently large, Bayes estimates of the model parameters appear to be close to the assumed value. When sample size is reduced, SDs of the β’s become larger, going from 0.2 to 0.45 as the sample size drops from 50 to 10. However, estimation itself seems to be unbiased, even in the case of only 10 replicates. In the second example in Table 5, one parameter was set to zero, providing a case where there is no linkage from gene 1 to gene 3. In this case, we see that the estimation for a nonexistent link is approximately zero, and one could discard this link. Similar results were seen for all the cases studied.

To further challenge the estimation procedure, an eight-gene network was simulated and estimated. This network had 18 parameters and was a greater challenge to the Bayesian method. Because of the increase in the number of parameters, SDs were substantially larger than in the four-gene model, but the estimation was still effectively unbiased.

## Discussion

Many methods have been developed for the analysis of gene expression microarray data, but few methods exist for using these data to quantify the interrelated behavior of genes within gene interaction networks. Most network-based methods are focused on network identification, not quantification. Given a hypothesized gene interaction network, this article develops and demonstrates the use of Bayesian network models as a tool for the analysis of a network using microarray data. The method allows for evaluating the strength of relationships within a hypothesized network and could also be used to test for additional linkages within the network.

There were two key points raised by these analyses. First, the application of this quantitative approach to the experimental data on TCDD effects in human lung epithelial cells clearly identified two subnetworks as significantly related to the AhR battery and the retinoid signaling. This indicates that the observed gene expression changes are consistent with the underlying hypothesized mechanism of action. In one sense this represents an alternate validation step in a tiered approach to evaluating microarray analyses. For example, *ZNF42*, although annotated as a retinoid-responsive gene, has not been previously validated as a retinoid-responsive gene in this cell system. The quantitative modeling suggests a highly significant relationship between *ZNF42* expression and other genes in the retinoid-signaling subnetwork, which provides confidence that its alteration was indeed due to activation of the retinoid-signaling pathway. The testing of other subnetworks within a given data set can further serve to increase confidence that inferences on relationships between genes obtained from other types of analyses (evaluation of gene annotation, clustering, pathway analysis, informatic-based network mapping, literature searches) are real.

The second point from these analyses is that we were able to test the interaction between the two subnetworks (AhR and retinoid) and illustrated that a functional relationship was likely real. Such an analysis is useful in that it supports further testing of this mechanism experimentally. It may be that some fraction of the toxicity associated with chronic exposure to TCDD could be the direct result of TCDD-induced increases in RA in the cells. The hypothesized network clearly supports a significant change in gene expression associated with signaling through the *RARB* pathway. The quantitative linkages observed in this experiment are unlikely to hold for an *in vivo* system but suggest that an experiment exposing laboratory animals to TCDD, which includes both TCDD and RA measurements with gene expression measurements, would be useful. Two recent experiments address these issues to a limited extent. Schmidt et al. (2003) examined RA levels and changes in expression of *CRBP1* in male Sprague-Dawley rats and saw significant changes in RA levels in kidney, liver, and serum, and a marginal change in liver *CRBP1* after 28 days. They did not examine any of the genes in the network shown in Figure 2, so it is difficult to compare directly with our results. Johnson et al. (2004) used *in vitro* data from three experiments with AhR ligands activating genes in the heart, kidney, and thoracic aorta of mouse embryos. They used an exhaustive search of three linkages for each gene to identify the most likely gene–gene interactions. They also identified linkages to genes in the RA-signaling pathway (*IGFBP-3* and *IGFBP-6*), but again, not the specific genes used in Figure 2.

The simulation experiments were different from the analysis of the TCDD study. In the TCDD study, the network linkages were perturbed to cause significant quantitative changes in expression, which then could be used to quantify the linkages between genes. In contrast, the simulation study used only the random variation in expression levels to quantify the network. The simulation studies indicate that the proposed method appears to be unbiased and, on average, produces the correct results. However, sample size could be a problem for small experiments with minor changes in gene expression. When the sample size is only 10 microarrays, the SD can be large relative to the expected value of the linkage between two genes, suggesting one might misinterpret a linkage as having little statistical support. This problem gets worse as the number of genes in the network increases. In contrast, large sample sizes of 50 microarrays are unlikely to have this problem.

Directed changes in the network, as in the dioxin experiment, can help overcome this problem and allow the quantification of significant linkages by as few as nine microarrays. To address this question, two additional simulations were conducted. Using the network shown in Figure 2 and the parameters estimated for the TCDD network shown in Table 3, we simulated 500 data sets consisting of nine microarrays—three for each dioxin dose; that is, we replicated the experiment 500 times using the predicted model. On average the resulting parameter estimates were identical to those observed from fitting the original data but appeared to have a slightly smaller SD than that estimated in the model. This decrease in SD could indicate a degree of model misspecification, as the simulated data appear to fit better than the observed data. In addition, whereas the observed data showed a nonsignificant linkage between *CYP1A1* to *RARB*, 48% of the simulated data sets found this linkage to be significant. Similarly, 54% found the linkage between *ALDH6* and *RARB* to be significant. In contrast, the simulations found a significant linkage between *NCOA2* and *ZNF42* in only 6% of the cases (hence the Type I error appears to be good) and between TCDD and *CYP1A1* in 100% of the cases (power is high).

In a second simulation, the network shown in Figure 2 was again simulated, this time without TCDD included in the experimental design and using just random variation in the genes to produce the data. Again, the results were unbiased, but the SDs more than doubled. In addition, the probability of observing a significant linkage was reduced by about 20% for most linkages. This illustrates the value of stimulating the system when trying to identify gene interaction networks.

Clearly, this type of modeling approach is limited in terms of interpretation. First, the model cannot be cyclic; hence, increases in *CRABP* as a function of *RARB* that might then result in greater binding of RA in the cytosol, reducing *RARB* expression, could not be included. Given time-course data, it could be possible to explore this linkage using a more complicated modeling form or some other method of analysis such as semicyclic Bayesian networks. Second, the method is dependent on a parametric model, and the choice of this model could impact the overall findings from the analysis. For example, if certain genes reached their maximal expression at lower doses of TCDD, the use of a log-linear model could underestimate low-dose changes while overestimating high-dose changes. This, in turn, could lead one to accept or reject a given model incorrectly. It should be noted that this type of criticism applies to all the other network analysis methods as well. Finally, although not seen in this analysis, it is possible that the resulting distributions for the linkages between the genes could be sensitive to the choice of prior distributions, and one should be careful to evaluate if such an impact might exist with the data.

Although the approach presented here involves only gene expression data, it can easily be expanded to include other data relevant to the linkages between genes and the quantification of signal transduction pathways in cells. Data quantifying protein levels in cells could easily be folded into a general likelihood, linked via a similar model, and analyzed to quantify the entire network. Such an approach leads to rational, mechanism-driven simultaneous analyses of genomics, proteomics, and metabolomics data. In addition, the networks identified through this type of analysis can easily be combined with other mechanism-based mathematical models such as physiologically based pharmaco-kinetic and pharmacodynamic models to present a true, systems-biology approach for the quantification of risks from exposures to xenobiotics like dioxin. This analysis would form one module of an overall model for TCDD toxicity. For example, if microarray data were available in rats exposed to TCDD, existing models like that of Kohn et al. (2001) could easily be linked to the gene interaction network discussed above. These, in turn, could be linked to cancer data using a mechanistic model to test hypotheses regarding cancer incidence and the mechanisms involved, as shown by Brooks et al. (1999).

The method proposed here is not restricted to the log-linear model used in this analysis, nor is it linked to the statistical likelihood chosen for the analysis. Other models such as dynamic models (Chen et al. 1999) and other statistical likelihoods (Wolfinger et al. 2001) could easily be incorporated into the analysis methods.

Bayesian networks have been used in a number of settings to provide insight into the complicated linkage between variables that interact. Quantifying the distributions linking genes into networks and expanding this to include proteins and protein modifications will make it possible to quantify the impact of a given chemical agent on the signal transduction pathways in a cell. Although many different methods could be used for this, Bayesian networks have the advantage of flexibility, which will make it possible to build on existing knowledge while bringing new data into the analysis. For the dioxin study presented here, the limitations of the sample size preclude an overall conclusion concerning the validity of the final model for predictions about the role of dioxin in changes to the RAR-signaling pathway. However, this analysis has strengthened the underlying hypothesis that changes in RAR signaling may play an important role in dioxin-mediated toxicity and suggest a number of experiments that could lead to a better-characterized network; this is left for future work.

In this article we used known scientific inferences and gene annotation to develop the initial tested network. This approach can also be applied to evaluating the likelihood of any hypothesized network developed by other approaches. As such, it can be applied to networks developed using other types of analyses including Bayesian, Boolean, and informatics-based approaches, as well as other known networks in the scientific literature. The ability to test hypotheses in the context of the network and to build modules that can be quantitatively linked to toxicity are first steps in a true systems-biology approach to mechanism-based use of genomics in risk assessment. This analysis is unique in that it directly addresses these uses.

## Figures and Tables

**Table 1:** Description of genes included in the gene interaction network shown in Figure 2.

Gene symbol (alternate symbols)*^a^*	Accession no.*^a^*	Gene name*^a^*	Biological role
*ALDH3 (ALDH3A1)*	AA069024	Aldehyde dehydrogenase 3 family, memberA1	May play a role in the oxidation of lipid aldehydes, especially those generated by lipid peroxidation (Vasiliou et al. 2000); is induced in rat liver by TCDD (Unkila et al. 1993)
*ALDH6 (ALDH1A3)*	AA054748	Aldehyde dehydrogenase 1 family, member A3	Has ability to synthesize retinoic acid from both retinol and retinal (Rexer et al. 2001)
*ALDH10 (ALDH3A2)*	H63779	Aldehyde dehydrogenase 3 family, member A2	Oxidizes long-chain aliphatic aldehydes to fatty acid
*CYP1A1*	AA418907	Cytochrome P450, subfamily I, polypeptide 1A	Phase I enzyme; its expression is controlled by the AhR. Metabolically activates procarcinogens to genotoxic electrophilic intermediates (Nebert et al. 1996)
*CRABP*	N23941	Cellular retinoic acid binding protein 1	Small intracellular protein that is a carrier for RA (vitamin A)
*NCOA2 (SRC-2, TIF2, GRIP1)*	R77770	Nuclear receptor coactivator 2	Transcription coactivator of retinoid/thyroid receptors; a histone acetyltransferase that plays an important role in lipid metabolism and energy balance (Picard et al. 2002; Xu and Li 2003)
*RARB*	W93713	Retinoic acid receptor, beta	Hetero/homodimers associated with oncogenicity (Lin and Evans 2000); overexpression in oral squamous carcinoma cell lines; leads to growth arrest and apoptosis (Hayashi et al. 2001)
*CDKN1A (p21, Cip1)*	N23941	Cyclin-dependent kinase inhibitor 1A	Functions as a regulator of cell-cycle progression; overexpression linked to carcinogenesis (Biankin et al. 2001)
*ZNF42 (MZF1, MZF-1, MZF1B)*	R83364	Zinc finger protein 42	Transcription factor that belongs to the Kruppel family of zinc finger proteins; RA-responsive; plays a role in cell proliferation (Hromas et al. 1991)
*ELF3 (ESX, ESE1)*	H27939	E74-like factor 3 (ets domain transcription factor, epithelial-specific)	Transcription factor that transactivates genes involved in epithelial differentiation and host defense and mediators of proinflammatory responses (e.g., Socs3, Cebp/delta, Bcl3, and CC/CXC chemokines) (Mysorekar et al. 2002; Yoshida et al. 2000)
*ACOX1 (ACOX, PALMCOX)*	AA040205	Human peroxisomal acyl-CoA oxidase	First enzyme of the fatty acid β-oxidation pathway (Varanasi et al. 1994); changes in this gene are likely to affect endogenous levels of fatty acids known to activate the retinoic X receptor, thereby modulating gene expression (Issemann et al. 1993)

a From the NCBI (National Center for Biotechnology Information) Unigene database (http://www.ncbi.nlm.nih.gov/entrez/query.fcgi?db=unigene).

**Table 2:** Relative expression level (to control) of genes in the HPL1A cells exposed in replicate to three different concentrations of TCDD.*^a^*

Genes									
*ALDH10*	1.56	1.33	1.24	1.42	1.56	1.40	1.69	1.47	1.25
*ALDH3*	2.10	2.09	2.34	3.88	2.94	4.09	3.11	3.91	3.76
*ALDH6*	2.42	2.00	1.77	3.40	4.12	3.37	3.76	4.60	3.66
*CRABP*	0.63	0.69	0.74	0.51	0.48	0.47	0.29	0.46	0.41
*CDKN1A*	1.56	1.16	1.49	1.30	1.34	1.58	1.51	1.49	1.63
*CYP1A1*	3.07	2.63	1.31	14.45	6.85	6.09	15.35	14.91	8.08
*ELF3*	1.56	1.37	1.18	2.19	1.70	1.91	3.15	2.00	2.02
*NCOA2*	1.42	1.41	0.82	1.34	1.07	0.92	1.42	1.22	0.82
*RARB*	1.64	1.42	0.93	1.77	1.56	1.21	1.48	1.63	1.15
*ZNF42*	1.88	1.47	1.11	1.62	1.43	1.32	1.60	1.45	1.22
*ACOX1*	1.94	1.50	0.78	1.93	1.03	0.84	10.87	1.22	0.59
TCDD*^b^*	0.10	0.10	0.10	1.00	1.00	1.00	10.0	10.0	10.0

a Data from Martinez et al. (2002).

b TCDD dose unit is measured in nanomolars. Actual doses are used for TCDD in the analysis.

**Table 3:** Type of linkage, mean, SD, and percentage of the posterior distribution below zero for all gene–gene relationships in Figure 2.

From	To	Type	Mean	SD	% < 0
TCDD	*ALDH3*	A	0.140	0.037	0.03
	*ALDH6*	A	0.150	0.035	0.01
	*ALDH10*	A	0.041	0.013	0.23
	*CYP1A1*	A	0.269	0.056	0.003
*ALDH6*	*CRABP*	R	0.348	0.152	1.27
	*NCOA2*	R	0.132	0.062	2.10
	*RARB*	A	0.149	0.191	19.81
*CYP1A1*	*CRABP*	R	0.235	0.099	1.03
	*NCOA2*	R	0.046	0.038	11.40
	*RARB*	A	0.072	0.120	26.34
*NCOA2*	*CDKN1A*	R	0.847	0.298	0.50
	*ZNF42*	A	0.000	0.168	48.53
*RARB*	*CRABP*	A	0.418	0.234	3.13
	*CDKN1A*	A	1.186	0.199	0.00
	*ELF3*	A	1.423	0.220	0.00
	*NCOA2*	A	0.912	0.085	0.00
	*ZNF42*	A	0.975	0.113	0.00
*ACOX1*	*NCOA2*	A	0.125	0.021	0.00
	*RARB*	A	0.081	0.065	9.38

Abbreviations: A, activate; R, repress.

**Table 4:** Estimated mean and median SD (σ) for genes included in the gene interaction network shown in Figure 2.

	Posterior distribution for σ	
Genes	Mean (median)	SD
*ALDH10*	0.22 (0.22)	0.04
*ALDH3*	0.63 (0.61)	0.12
*ALDH6*	0.62 (0.61)	0.12
*CRABP*	0.11 (0.11)	0.02
*CDKN1A*	0.15 (0.14)	0.03
*CYP1A1*	0.94 (0.92)	0.17
*ELF3*	0.25 (0.24)	0.05
*NCOA2*	0.04 (0.04)	0.01
*RARB*	0.13 (0.12)	0.03
*ZNF42*	0.08 (0.08)	0.02
*ACOX1*	0.86 (0.82)	0.20

**Table 5:** Mean, median, and SD from two simulation studies of the simple four-gene model (Figure 1).

Model	Sample size	Estimation	β_14_	β_13_	β_23_	β_34_	σ_1_	σ_2_	σ_3_	σ_4_
β_14_ = −2	50	Mean (SD)	−1.98 (0.22)	0.81 (0.21)	0.83 (0.19)	−1.32 (0.13)	1.01 (0.12)	1.00 (0.12)	1.03 (0.12)	1.03 (0.13)
β_13_ = 0.8		Median (SD)	−1.97 (0.22)	0.81 (0.21)	0.82 (0.19)	−1.33 (0.13)	1.01 (0.13)	1.00 (0.12)	1.02 (0.13)	1.02 (0.14)
β_23_ = 0.8	25	Mean (SD)	−2.00 (0.29)	0.80 (0.25)	0.81 (0.23)	−1.29 (0.19)	1.05 (0.15)	1.03 (0.15)	1.05 (0.16)	1.06 (0.16)
β_34_ = −1.3		Median (SD)	−1.98 (0.29)	0.80 (0.26)	0.78 (0.24)	−1.30 (0.19)	1.02 (0.15)	1.01 (0.15)	1.02 (0.16)	1.03 (0.17)
σ_*i*_ = 1, *i* = 1,2,3,4	10	Mean (SD)	−1.97 (0.45)	0.79 (0.40)	0.80 (0.38)	−1.29 (0.29)	1.13 (0.26)	1.10 (0.27)	1.15 (0.32)	1.19 (0.29)
		Median (SD)	−1.95 (0.45)	0.79 (0.40)	0.81 (0.37)	−1.31 (0.29)	1.08 (0.24)	1.04 (0.26)	1.04 (0.31)	1.11 (0.28)
β_14_ = −2	50	Mean (SD)	2.00 (0.17)	0.01 (0.17)	0.80 (0.17)	−1.30 (0.12)	1.02 (0.11)	1.03 (0.12)	1.04 (0.12)	1.01 (0.12)
β_13_ = 0		Median (SD)	2.0 (0.18)	0.01 (0.18)	0.81 (0.18)	−1.31 (0.13)	1.01 (0.12)	1.03 (0.12)	1.04 (0.14)	1.00 (0.13)
β_23_ = 0.8	25	Mean (SD)	2.0 (0.22)	0.01 (0.22)	0.79 (0.21)	−1.31 (0.16)	1.04 (0.15)	1.05 (0.15)	1.06 (0.16)	1.04 (0.17)
β_34_ = −1.3		Median (SD)	2.01 (0.23)	0.00 (0.23)	0.77 (0.21)	−1.30 (0.16)	1.02 (0.15)	1.03 (0.15)	1.03 (0.16)	1.03 (0.17)
σ_*i*_ = 1, *i* = 1,2,3,4	10	Mean (SD)	2.02 (0.40)	−0.02 (0.40)	0.83 (0.40)	−1.30 (0.32)	1.14 (0.25)	1.13 (0.27)	1.16 (0.30)	1.18 (0.26)
		Median (SD)	1.99 (0.40)	−0.02 (0.39)	0.85 (0.39)	−1.29 (0.31)	1.08 (0.24)	1.06 (0.27)	1.10 (0.30)	1.10 (0.25)
